# Optimizing multiomics sample preparation: comparative evaluation of extraction protocols for HepG2 cells

**DOI:** 10.1007/s00216-025-06235-x

**Published:** 2025-11-19

**Authors:** Tilman F. Arnst, Selina Hemmer, Claudia Fecher-Trost, Lea Wagmann, Markus R. Meyer

**Affiliations:** Department of Experimental and Clinical Toxicology and Pharmacology, Institute of Experimental and Clinical Pharmacology and Toxicology, Center for Molecular Signaling (PZMS), PharmaScienceHub (PSH), Saarland University, Homburg, Germany

**Keywords:** Multiomics, Sample preparation, HepG2 cells, Metabolomics, Proteomics, Mass spectrometry

## Abstract

Multiomics approaches enable a comprehensive characterization of complex biological systems by simultaneously investigating multiple molecular layers. Generating multiple omics datasets from a single sample is crucial to minimize biological variability and ensure cross-layer consistency, which is critical for robust downstream data analysis. However, existing workflows often require adaptation to the specific experimental context and instrumental setup. This study systematically compared two established protocols for the simultaneous extraction of metabolites, lipids, and proteins from HepG2 cells: (i) a biphasic extraction with subsequent overnight protein digestion from the interphase pellet, and (ii) a monophasic extraction involving on-bead protein digestion. For the monophasic approach, we further investigated the effects of bead size and digestion conditions. Metabolomics samples were analyzed using liquid chromatography coupled to high-resolution tandem mass spectrometry; lipidomics and proteomics samples were analyzed by nano-scale liquid chromatography coupled with ion mobility separation and high-resolution tandem mass spectrometry. Each method was evaluated in terms of total feature count, selectivity, reproducibility, handling complexity, and overall performance. While neither protocol was optimal across all criteria, the monophasic extraction using paramagnetic beads with shortened incubation time proved to be the most reproducible, efficient, and cost-effective solution for in-house multiomics workflows in HepG2 cells.

**Supplementary Information:**

The online version contains supplementary material available at 10.1007/s00216-025-06235-x.

## Introduction

Advances in single-omics technologies have deepened our understanding of biological systems at the molecular level. Approaches such as metabolomics, lipidomics, and proteomics have allowed critical insights into specific pathways and regulatory mechanisms across diverse areas of research. Untargeted metabolomic workflows have become indispensable for uncovering previously unknown biomarkers and characterizing metabolic shifts driven by e.g., environmental factors or drug exposure [1]. Lipidomics has undergone considerable development, with enhanced structural sensitivity facilitating detailed investigations of lipid signaling, membrane architecture, and cell-type-specific lipid distributions [2]. Although not widely applied yet in metabolomics and lipidomics, the implementation of nanoflow liquid chromatography holds great promise by further enhancing analytical sensitivity [3]. Bottom-up proteomics has been used for decades for cataloging proteins expressed in cells and tissues. Recent technological improvements have enabled detailed analysis of post-translational modifications and quantitative proteomics approaches [4]. Furthermore, single‐pot solid‐phase‐enhanced sample preparation has enabled high-throughput and streamlined workflows for large clinical investigations [5–8].

Despite these significant advances in individual methods, focusing solely on a single-omics layer is often insufficient for unraveling biological complexity, as crucial regulatory interactions at the molecular level may be overlooked [9, 10]. This limitation has driven a transition toward integrated molecular profiling within systems biology, where multiomics approaches have emerged as a powerful strategy to integrate genomics, transcriptomics, proteomics, lipidomics, and metabolomics information [9–15]. To minimize confounding effects due to biological variability and allow comprehensive analyses, it is crucial to generate multiple omics datasets from the same sample, whenever possible.

Several integrative protocols allowing simultaneous untargeted metabolomics, lipidomics, and proteomics analysis from diverse sample types (such as tissues, biofluids, and cells) have been developed [16–20]. Established biphasic sample preparations for lipid analysis, e.g., Matyash et al.-derived methyl-*tert*-butyl ether (MTBE)-based extractions, were adapted for lipidomics and metabolomics analysis [21]. They enable the separation of polar metabolites and lipids into two separate phases for metabolomics and lipidomics analysis. Subsequent proteomics analysis can be achieved after solubilization and digestion of the protein-containing interphase pellet [22]. Recently, Muehlbauer et al. developed a bead-enabled accelerated monophasic multiomics method for the concurrent extraction of metabolites and lipids, coupled with on-bead protein aggregation and accelerated short-term 40-min tryptic digestion to enable efficient proteomics analysis. While molecular coverage and data quality have been demonstrated for various sample types, including human plasma, pelleted cells, and tissues, its transferability to specific experimental conditions and instrumental setups has still to be investigated [23]. This is particularly important, as variations in the analytical setup—such as the liquid chromatography flow regime, the choice of column, and the use of ion mobility spectrometry—can significantly affect analytical depth, feature detection, and overall data interpretability [3, 24–26]. Consequently, evaluation and adaptation of established workflows to individual requirements must be performed. For the current study, HepG2 cells were used, as they represent a widely applied toxicological model for hepatoxicity screening and metabolism studies, among others [27–29]. Following the adaptation to a specific setup, including the implementation of nanoflow liquid chromatography ion mobility spectrometry high-resolution mass spectrometry (LC-IMS-HRMS) for lipidomics and proteomics analysis, the current study aimed to compare the suitability of a monophasic bead-based approach with a biphasic MTBE-based sample preparation procedure for integrated multiomics analysis of plated HepG2 cells. Additionally, modifications to the monophasic protocol, particularly in terms of bead size and enzymatic digestion conditions, were systematically investigated to assess their influence on data quality.

## Experimental section

### Chemicals and materials

Trypsin (trypsin gold, mass spectrometry grade) and rapid trypsin (rapid trypsin gold, mass spectrometry grade) were obtained from Promega (Walldorf, Germany). Silica-coated superparamagnetic beads SeraSil-Mag 400 and SeraSil-Mag 700 were purchased from Cytiva (Marlborough, MA, USA). Tris(2-carboxyethyl)phosphine hydrochloride (TCEP) and chloroacetamide (CAA) were bought from Thermo Scientific (Rockfold, IL, US). L-Tryptophan-d_5_ was obtained from Alsachim (Illkirch-Graffenstaden, France). L-Carnitine-d_9_, cytosine-d_2_, kynurenic acid-d_5_, stearic acid-^13^C, and thymidine-d_4_ were purchased from Cayman Chemical (Ann Arbor, MI, USA) and L-arginine-d_7_ (L-arginine- 2,3,3,4,4,5,5-d_7_) from Toronto Research Chemicals (Toronto, Canada). *N*-Pentadecanoyl-D-erythro-sphingosine(d_7_), 1-pentadecanoyl-2-oleoyl(d_7_)-sn-glycero-3-phosphocholine, and phosphoric acid (85%) were obtained from Merck (Darmstadt, Germany). Acetonitrile (ACN), ammonium formate, ammonium acetate, *n*-butanol, ESI-L Low Concentration Tuning Mix, formic acid, isopropanol (IPA), methanol, methyl-*tert*-butyl ether, Pierce™ C18 pipette tips (10 µL), trifluoroacetic acid, nanofiltered water (filtered at 0.2 µm), and all other chemicals (LC-MS or analytical grade) were purchased from VWR International (Darmstadt, Germany). Fetal bovine serum (FBS) was purchased from Corning (Amsterdam, The Netherlands). RPMI 1640 medium with GlutaMAX supplement was obtained from Life Invitrogen (Darmstadt, Germany). HepG2 cells were provided by the German Collection of Microorganisms and Cell Cultures (DSMZ, Braunschweig, Germany). Single stocks were cryopreserved and stored in liquid nitrogen at −160 °C until use.

### Cell culture

HepG2 cells were cultured at 37 °C in a humidified atmosphere containing 5% CO_2_ with RPMI 1640 medium containing 10% FBS, 100 U/mL penicillin, and 100 μg/mL streptomycin as previously described. Passaging was performed every 3–4 days using PBS (137 mM NaCl, 2.7 mM KCl, 1.5 mM KH_2_PO_4_, 8.1 mM Na_2_HPO_4_, pH 7.4) and 0.05% trypsin EDTA solution [30]. Cells were seeded in 24-well plates (250.000 per well) for 48 h with growth medium before the medium was changed for another 24 h. Afterwards the supernatant was discarded, cells were washed with PBS, and the plates were stored at −80 °C until sample preparation.

### Sample preparation

Details on the number of replicates for each extraction and digestion condition are provided in Table S1 of the Supplementary Information.

#### Monophasic extraction and on-bead digestion

Samples were prepared according to a previously published procedure by Muehlbauer et al. [23] as follows. HepG2 cells seeded in 24-well plates were placed on ice, and 420 µL of ice cold (−20 °C) *n-*butanol:ACN (3:1, *v:v*) containing eight isotope-labeled endogenous compounds as internal standards (Table S2) was added. Efficient cell detachment and lysis were ensured by mechanical scratching and shear forces generated by pipetting the suspension up and down 20 times. Bead stock solutions were freshly prepared according to Supplementary Information; 80 µL containing either 400 nm or 700 nm unmodified silica beads was added, resulting in a final water percentage of approximately 20% and a bead-to-protein ratio of 10:1. The lysate was transferred into a reaction tube, vortexed, sonicated in a chilled water bath for 5 min prior to incubation on ice for 5 min, and placed on a magnetic rack for 30 s.

For metabolomics and lipidomics analysis, 225 µL of the resulting supernatant was aliquoted into separate MS vials. After evaporation to dryness using a vacuum centrifuge at 1400 rpm and 24 °C, samples were stored at −20 °C for up to 2 weeks until analysis. For metabolomics analysis, the obtained residues were reconstituted in 60 µL ACN:water (1:1, *v:v*), whereas for lipidomics analysis, 30 µL *n*-butanol:IPA:water (8:23:69, *v:v:v*) with 5 mM phosphoric acid was added as described in the literature [3]. For each analysis, pooled quality control samples (QC) were prepared by transferring equal proportions of each sample into one MS vial.

For proteomics analysis, four different on-bead digestions were assessed per bead type (400 nm or 700 nm unmodified silica beads). Detailed digestion conditions are specified in Table 1. Therefore, the resulting bead residue was resuspended in protein digestion buffer (Promega) and vortexed. A volume containing 100 µg of total protein estimated based on BCA assay results (described in the Supplementary Information) was transferred to a new reaction tube. Subsequently, the reaction tube was placed on a magnetic rack for 30 s. For accelerated on-bead digestions, the supernatant was replaced with 100-µL digestion buffer spiked with 5 mM TCEP, 20 mM CAA, and various amounts of rapid trypsin (specified in Table 1). For overnight on-bead digestion, the supernatant was replaced with 100 µL 50 mM Tris spiked with 10 mM TCEP, 40 mM CAA, and trypsin (enzyme-to-protein ratio 1:50). Afterwards, all samples were incubated at 1000 rpm. Following digestion, samples were incubated on a magnetic rack for 30 s; the supernatant was recovered and acidified with 50 µL 2.5% trifluoroacetic acid. Peptides were desalted and purified with Pierce™ C18 pipette tips according to the manufacturer’s instructions, evaporated to dryness at 1400 rpm and 24 °C, and stored at −20 °C for up to 2 weeks until analysis. Residues were reconstituted in 20 µL 0.1% formic acid in water prior to analysis.

**Table 1:** Overview of investigated sample preparation procedures for metabolomics, lipidomics and proteomics analysis, indicating bead diameter, enzyme type, E:P (enzyme-to-protein ratio), incubation time and temperature for the associated tryptic digestion for proteomics analysis. MTBE (methyl-*tert*-butyl ether), ME (monophasic extraction), OV (overnight, 840 min)

Sample preparation		Bead diameter	Enzyme	E:P	Incubation time	Temperature
Monophasic extraction (ME)	ME 400_40	400 nm	Rapid trypsin	1:10	40 min	60 °C
	ME 400_60	400 nm	Rapid trypsin	1:25	60 min	60 °C
	ME 400_90	400 nm	Rapid trypsin	1:25	90 min	60 °C
	ME 400_OV	400 nm	Trypsin	1:50	840 min	23 °C
	ME 700_40	700 nm	Rapid trypsin	1:10	40 min	60 °C
	ME 700_60	700 nm	Rapid trypsin	1:25	60 min	60 °C
	ME 700_90	700 nm	Rapid trypsin	1:25	90 min	60 °C
	ME 700_OV	700 nm	Trypsin	1:50	840 min	23 °C
Biphasic extraction	MTBE-based	-	Trypsin	1:50	840 min	23 °C

#### Biphasic MTBE-based extraction and interphase pellet protein digestion

According to previously published procedures [21, 23], in 24-well plates, seeded HepG2 cells were placed on ice and 420 µL ice cold (−20 °C) MeOH:MTBE (3:10, *v:v*) containing eight internal standards (Table S2) was added. HepG2 cells were detached and lysed as described above. The resulting suspension was transferred into a reaction tube, vortexed, and sonicated in a chilled water bath for 5 min. The suspension was incubated on ice for 1 h at 1400 rpm. Afterwards, phase separation was induced by adding 80 µL water resulting in a final composition of water:MeOH:MTBE (2.5:3:10, *v:v:v*). After incubation on ice for 10 min, samples were centrifuged at 15,000 × *g* at 4 °C for 5 min. The upper organic and the lower aqueous layer were transferred into separate MS vials for lipidomics and metabolomics analysis, respectively. Evaporation, storage, and reconstitution were performed as described in Section “Monophasic extraction and on-bead digestion.”

For proteomics analysis, the resulting protein pellet was washed with 50 µL ACN and evaporated in a vacuum centrifuge at 24 °C and 1400 rpm. Resolubilization was done by the addition of 50 µL resolubilization buffer (8 M urea, 10 mM TCEP, and 50 mM Tris, pH 8), and sonication in a chilled water bath for 7.5 min. Afterwards, the samples were diluted with digestion buffer (10 mM TCEP, 40 mM CAA and Tris 50 mM, pH 8) to a final concentration of 1 M urea. A volume containing 100 µg of total protein was estimated based on BCA assay results (described in the Supplementary Information) and transferred to a fresh reaction tube. Subsequently, trypsin was added in an estimated enzyme-to-protein ratio of 1:50. Digestion was performed on a shaker for 840 min at 1000 rpm and 23 °C as specified in Table 1. The reaction was terminated by acidification with trifluoroacetic acid to a final concentration of 1%. Peptides were desalted, evaporated, and stored until analysis as described above.

### LC-HRMS/MS and nano-LC-IMS-HRMS/MS apparatus

#### Metabolomics analysis using LC-HRMS/MS

Analysis was performed according to previously published studies [31–33] using a Thermo Fisher Scientific (TF, Dreieich, Germany) Dionex UltiMate 3000 RS pump consisting of a degasser, a quaternary pump, and an UltiMate Autosampler, coupled to a TF Q Exactive Plus high-resolution mass spectrometer equipped with a heated electrospray ionization (HESI)-II source. Performance of the columns and the mass spectrometer was tested using a test mixture described by Maurer et al. [34, 35]. For preparation and cleaning of the injection system, IPA:water (9:1, *v:v*) was used. Gradient elution was performed on a SeQuant ZIC HILIC column (3.5 µm, 150 mm × 2.1 mm) using a flow rate of 0.5 mL min^−1^. The mobile phase consisted of aqueous ammonium acetate (200 mM, eluent A) and ACN containing formic acid (0.1%, *v:v*, eluent B). The gradient was programmed as follows: 0.0 min 98% B, 0.0–5.0 min 80% B, 5.0–8.5 min 40% B, 8.5–10 min hold 40% B, 10–12 min 98% B. The injection volume was set to 2 µL and the column oven temperature was 40 °C. Mass spectrometry for untargeted metabolomics was performed using data-dependent acquisition mode. Details on MS settings are given in Table S3. All samples were analyzed in randomized order, to avoid potential analyte instability or instrument performance confounding data interpretation. In addition, one pooled QC injection was performed every sixth sample to monitor batch effects, as described by Wehrens et al. [36].

#### Lipidomics analysis using nano-LC-IMS-HRMS/MS

Analysis was performed using a nanoElute (Bruker Daltonics, Bremen, Germany) ultra-high-performance nanoflow chromatography system coupled with a CaptiveSpray source to a trapped ion mobility-quadrupole time-of-flight mass spectrometer (timsTOF Pro 2, Bruker Daltonics). Pre-acquisition mass and ion mobility calibration was performed according to the manufacturer’s instructions with ESI-L Low Concentration Tuning Mix. Gradient elution was done by reversed-phase chromatography using a PepMap™ Neo column (150 mm × 75 µm, TF Scientific), and lipid separation was performed using eluent A (ACN:water, 3:2, *v:v*) and eluent B (IPA:ACN, 9:1, *v:v*), both buffered with 10 mM ammonium formate and 0.1% formic acid (*v:v*) [24]. The gradient was programmed as follows: 0.0 min 1% B, 0.0–3.0 min 30% B, 3.0–7.0 min 51% B, 7.0–12 min 61% B, 12–17 min 71% B, 17–22 min 99% B, 22–27 min hold 99% B, 27–28 min 1% B, 28–30 hold 1% B. The injection volume was set to 2 µL and the column oven temperature was 40 °C. An injection volume of 2 µL was used for both polarities, the flow rate was set to 0.35 µL/min, and the column oven temperature was 60 °C. For the purpose of post-acquisition mass data recalibration, 1 mM sodium formate calibration solution was automatically injected at the end of each run. Mass spectrometry for untargeted lipidomics was performed using data-dependent acquisition mode. Details on MS settings are shown in Table S4 in the Supplementary Information. All samples were analyzed in randomized order. Additionally, one QC sample was performed every sixth sample as described above. Post-acquisition mass recalibration of all data files was performed with a high-performance calibration model with at least seven calibrants in Bruker Compass DataAnalysis (Version 6.1) prior to further processing.

#### Proteomics analysis using nano-LC-IMS-HRMS/MS

Proteomics analysis was performed on a nanoElute (Bruker Daltonics) coupled with a CaptiveSpray source to a timsTOF Pro 2 (Bruker Daltonics) [37]. Pre-acquisition mass and ion mobility calibration were performed as described above. Six-microliter samples were loaded onto a PepMap™ Neo Trap Cartridge (300 µm × 5 mm, TF Scientific) and separated using a Thermo PepMap™ Neo column (150 mm × 75 µm, TF Scientific). Peptide elution was performed at a flow rate of 0.40 µL min^−1^ using eluent A (water with 0.1% formic acid, *v:v*) and eluent B (ACN with 0.1% formic acid, *v:v*). The gradient was programmed as follows: 0.0 min 2% B, 0.0–30 min 17% B, 30–45 min 25% B, 45–50 min 37% B, 50–55 min 95% B, 55–62 min hold 95% B. The injection volume was set to 2 µL and the column oven temperature was 50 °C. Mass spectrometry for untargeted proteomics was performed using data-independent acquisition (DIA) mode. Details on MS settings are shown in Table S5 in the Supplementary Information. All samples were analyzed in randomized order. Post-acquisition lock mass recalibration of all raw files was performed in Bruker Compass DataAnalysis (Version 6.1) prior to further processing.

### Data processing and evaluation

The metabolomics and lipidomics data have been deposited to MetaboLights repository with the study identifier MTBLS12977 [38].

#### Metabolomics analysis

Untargeted metabolomics data processing was performed using MZmine (Version 4.5.19) [39, 40]. Thermo Fisher Scientific LC-HRMS/MS raw files were converted into mzML files using ProteoWizard [41]. MZmine processing parameters for mass detection, extracted ion chromatogram (EIC) building, EIC resolving, alignment, and gap-filling were manually optimized based on a representative number of samples prior to processing, as recommended in the literature [42]. Exact processing parameters are available as supplementary files via MetaboLights (MTBLS12977). Data from positive and negative ionization modes were processed separately. Each sample preparation procedure was processed independently using identical batch settings resulting in a separate feature list per sample preparation procedure. For the assessment of the total feature count, each feature list per sample preparation procedure was used, and the number of features with an assigned peak area was summed up for each analysis. For reproducibility, the coefficient of variation (CV) was determined from average intensity normalized peak areas. The number and proportion of features with CV < 20% were determined from separate feature lists for each sample preparation procedure. Furthermore, for a direct comparison, all sample preparation procedures were processed in a single batch and the CV distribution of selected features was assessed. Therefore, features were only selected, if they were initially aligned across all samples without gap-filling. Additionally, the separate feature lists were imported to SIRIUS (Version 6.1.1) [43]. Molecular formula prediction and ranking were achieved using the SIRIUS and ZODIAC algorithms [44], while CANOPUS was used for compound class prediction [45]. SIRIUS processing parameters are provided as Supplementary Information. As previously proposed by Brockbals et al. [46], the number of compounds classified as organic acids and derivatives (OAD) was used as evaluation criteria for untargeted metabolomics analysis. Furthermore, the ClassyFire SuperClass distribution was assessed. The prerequisite for both was a ZODIAC score > 0.8 and a ClassyFire superclass probability > 700.

#### Lipidomics analysis

Untargeted lipidomics analysis was evaluated by Metaboscape (version 2022b, Bruker Daltonics) using an integrated workflow that employs the T-ReX 4D algorithm for automatic retention time and ion mobility alignment, deisotoping, and feature extraction to generate a feature list. Exact processing parameters are available as supplementary files via MetaboLights (MTBLS12977). Mass recalibration was performed using sodium formate clusters in a retention time window of 30 to 40 min. The calculated ppm deviation was below 1 ppm using a high-performance calibration model with at least seven calibrants. Peak area normalization using probabilistic quotient normalization was applied [47]. Data evaluation was performed as described in Section “Metabolomics analysis,” and SIRIUS processing parameters are provided as Supplementary Information. In contrast to untargeted metabolomics analysis, the number of compounds classified as lipids and lipid-like species (LLLS) with a ZODIAC score > 0.8 and a ClassyFire SuperClass probability > 700 was considered [46]. Additionally, the ClassyFire class distribution of compounds classified as LLLS (lipids and lipid-like species) with a ClassyFire probability > 700 was assessed.

#### Proteomics analysis

Untargeted proteomics data were processed and quantified with PEAKS Studio (version 10.6 build 20201221, Bioinformatic Solutions Inc.), which leverages the additional ion mobility dimension and employs a hybrid de novo–database search strategy, resulting in enhanced sensitivity and identification accuracy. Peptide and protein identification was carried out with PEAKSLIB via monoisotopic precursor mass search with a parent mass error tolerance of 25.0 ppm and a fragment mass error tolerance of 0.05 Da. Identification was conducted using an in-house generated spectral library (201,675 peptide precursor entries), generated according to Supplementary Information, and the UniProt Homo sapiens database (20,354 sequences, Version November 2023) as the reference database. For peptide identification, three missed cleavages were allowed; a carbamidomethylation of cysteines was used as a static modification; and oxidation of methionine residues, acetylation of protein *N*-termini, and deamidation of asparagine and glutamine residues were allowed as variable modifications. For protein identification, at least two unique peptides with a false discovery rate (FDR) < 1% were required. Identification-directed label-free quantification was performed with the PEAKS Q module without outlier removal (ROUT) and CV filtering using a mass error tolerance of 20.0 ppm, an ion mobility tolerance (1/*k*_0_) of 0.05 v s/cm^2^, and a false discovery threshold of 1%. Retention time alignment was achieved based on an automatically detected retention time shift tolerance. Protein quantification was performed based on a minimum of two unique peptides which were detected in at least two samples per group. Significance testing was carried out using analysis of variance (ANOVA) on total ion count (TIC) normalized protein group abundances [48]. Only protein groups with *p*-value < 0.05 and a fold change > 2 were considered for further analysis. To analyze the distribution of proteins across the main Gene-Ontology (GO) cellular component categories (membrane GO 0016020; nucleus GO 0005634; cytoplasm GO 0005737), only proteins identified in all samples of the corresponding sample preparation procedures were considered. Uniprot-ID-mapping (Swissprot, uniport.org, https://www.uniprot.org/id-mapping) was used to retrieve the associated GO terms.

### Statistical analysis for total feature count, identified protein count, and reproducibility

Statistical evaluation for all three-omics analyses was performed in GraphPad Prism (Version 10.4.2), using ordinary one-way ANOVA with a Tukey’s post hoc multiple comparison test for total feature count or identified protein count comparisons. Therefore, the following settings were used: experimental design, no matching or paring; distribution assumption, normal (gaussian); family-wise alpha threshold and confidence level, 0.05 (95% confidence interval). For the comparison of the CVs of selected features (described in Section “Metabolomics analysis”), a robust regression and outlier removal (ROUT) [49] with a coefficient Q of 1% was applied prior to significance testing. Box plots of CVs range from the first quartile (25th percentile) to the third quartile (75th percentile) of distribution representing the interquartile range (IQR). Medians (50th percentiles) are indicated by the lines across the boxes. Whiskers range from minimum to maximum values.

## Results and discussion

### Metabolomics analysis

Comprehensive metabolomic profiling relies on the capability to detect a broad range of metabolites, to provide the most complete representation of the metabolome [50]. While the choice of the analytical technique plays a central role, the extent of metabolite detection is also strongly influenced by the sample preparation protocol, e.g., the selection of extraction and reconstitution solvent mixtures [51]. In LC-MS-based metabolomics, metabolite detection is typically represented by features, defined as a bounded, two-dimensional (*m/z* and retention time (RT) dimensions) signal characterized by a pair of *m/z* and RT values and associated with the detected signal intensity [52]. The total number of features detected is commonly used as a metric for comparing the performance of different sample preparation approaches in untargeted metabolomics studies [33, 50, 53, 54].

**Fig. 1:**
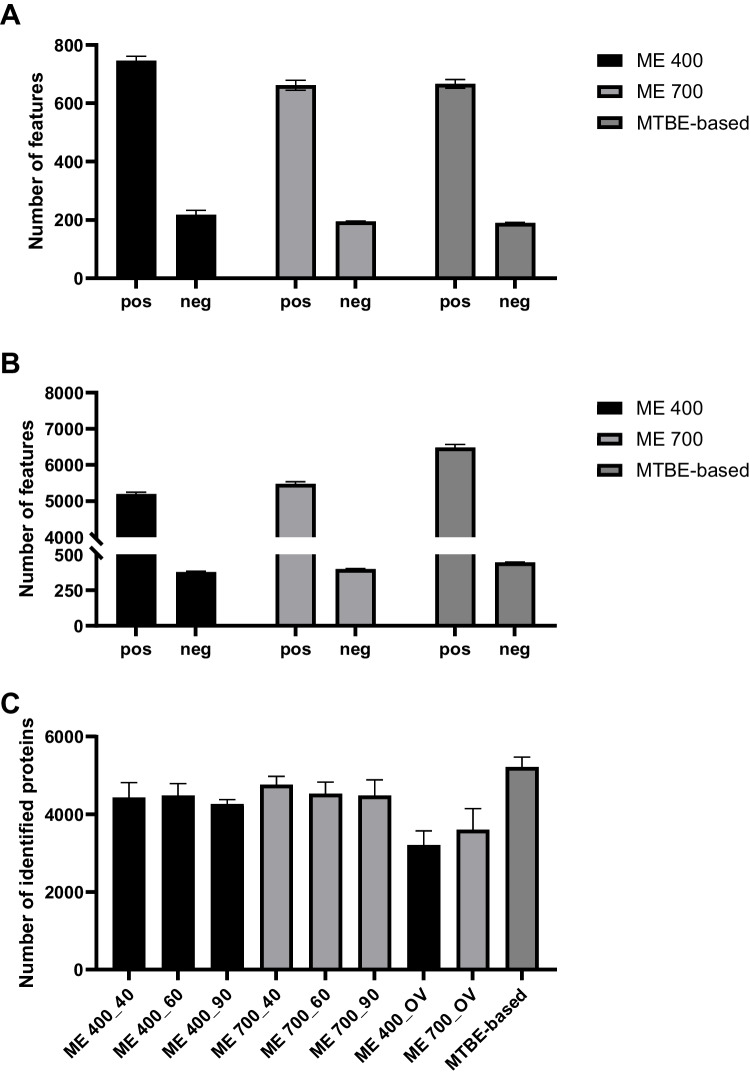
**A** Histograms of the number of features for metabolomics analysis detected in positive (pos) and negative (neg) ionization mode applying hydrophilic interaction chromatography for ME (Monophasic extraction) 400 (400 nm beads, *n* = 16), ME 700 (700 nm beads, *n* = 16), and MTBE (methyl-*tert*-butyl ether)-based (*n* = 8) sample preparation procedures. **B** Histograms of the number of features for lipidomics analysis detected in positive (pos) and negative (neg) ionization mode applying reversed-phase chromatography for ME 400 (*n* = 16), ME 700 (*n* = 16), and MTBE-based (*n* = 8) sample preparation procedures. **C** Histograms of the number of identified proteins for proteomics analysis in positive ionization mode applying reversed-phase chromatography ME 400, ME 700, and MTBE-based, 40 (40 min), 60 (60 min), 90 (90 min), and OV (overnight, 840 min). MTBE-based (*n* = 8), all others (*n* = 4). Bars and error bars indicate mean ± SD (standard deviation)

**Fig. 2:**
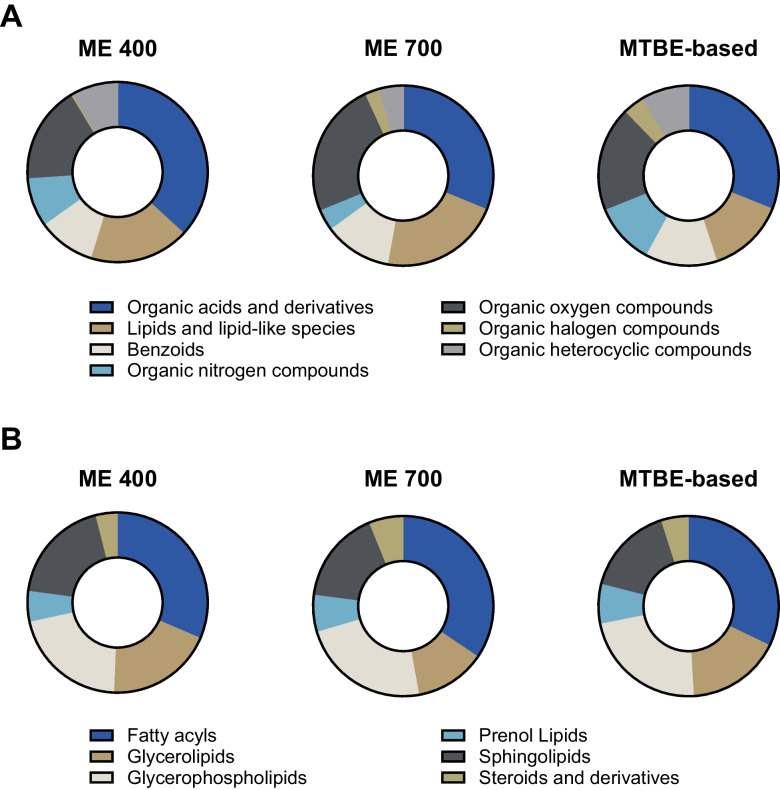
**A** Comparison of organic compound distribution obtained using ME (monophasic extraction) 400 (400 nm beads), ME 700 (700 nm beads), and MTBE (methyl-*tert*-butyl ether)-based extraction. Donut charts represent the relative proportion of organic compounds across the associated ClassyFire SuperClasses (benzoids, lipids and lipid-like species, organic acids and derivatives, organic halogen compounds, organic heterocyclic compounds, organic nitrogen compounds, organic oxygen compounds) with a ZODIAC score > 800 and a ClassyFire SuperClass probability > 700 using the CANOPUS and CSI:FingerID algorithm within SIRIUS (Version 6.1.0). **B** Comparison of lipid class distribution obtained using ME 400, ME 700, and MTBE (methyl-*tert*-butyl ether)-based extraction. Donut charts represent the relative proportion of lipids and lipid-like species (LLLS) across the associated ClassyFire classes (fatty acyls, glycerolipids, glycerophospholipids, prenol lipids, sphingolipids, steroids and derivatives) with a ZODIAC score > 800, a ClassyFire SuperClass probability > 700, and ClassyFire Class probability > 700 using the CANOPUS and CSI:FingerID algorithm within SIRIUS (Version 6.1.0)

**Fig. 3:**
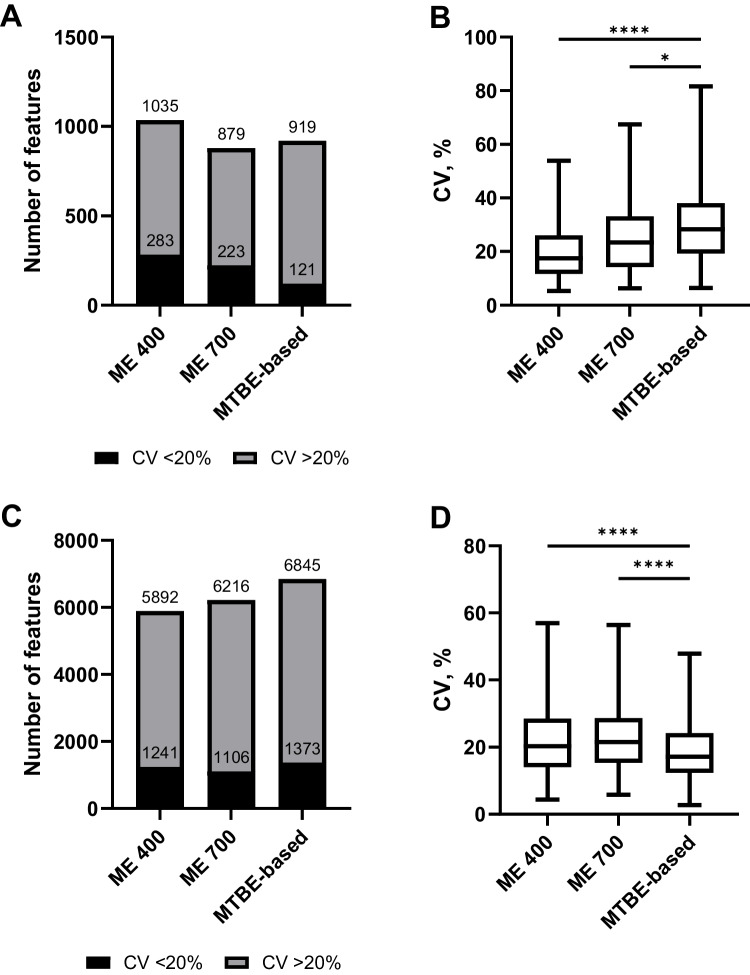
**A** Histograms of the number of features from metabolomics analysis applying positive and negative ionization mode with a normalized peak area CV < 20% (black) and > 20% (gray) assessed from separate feature lists per sample preparation procedure. **B** Box plots show the coefficient of variation (CV) distribution of normalized peak areas of 84 selected features aligned in all samples from positive and negative ionization mode from metabolomics analysis. Boxes represent the interquartile range (IQR), from the first quartile (25th percentile) to the third quartile (75th percentile). Medians (50th percentiles) are shown as horizontal lines within the boxes. Whiskers indicate the minimum and maximum values. Statistical evaluation using a one-way ANOVA with a Tukey’s post hoc multiple comparison test after robust regression and outlier removal (ROUT) with a coefficient Q of 1% (*p < 0.05; ****p < 0.0001). **C** Histograms of the number of features from lipidomics analysis applying positive and negative ionization mode with a normalized peak area CV < 20% (black) and > 20% (gray) assessed from separate feature lists per sample preparation procedure. **D** Box plots show the coefficient of variation (CV) distribution of normalized peak areas of 1718 selected features aligned in all samples from positive and negative ionization mode from lipidomics analysis. Boxes represent the interquartile range (IQR), from the first quartile (25th percentile) to the third quartile (75th percentile). Medians (50th percentiles) are shown as horizontal lines within the boxes. Whiskers indicate the minimum and maximum values. Statistical evaluation using a one-way ANOVA with a Tukey’s post hoc multiple comparison test after robust regression and outlier removal (ROUT) with a coefficient Q of 1% (****p < 0.0001). ME (monophasic extraction) 400 (*n* = 16), ME 700 (*n* = 16), and MTBE (methyl-*tert*-butyl ether)-based (*n* = 8) sample preparation procedures

Figure 1A illustrates the total feature count of the evaluated protocols. ME 400 resulted in the highest number of features for positive (745 ± 10) as well as negative ionization mode (221 ± 2). ME 700 and the MTBE-based procedure showed similar results regarding the total feature count for both ionization modes, although they were significantly lower compared to ME 400, as shown in Fig. 1A. As already discussed in the literature, an evaluation solely based on the total feature count is often insufficient as the fraction of (false positive) features lacking in biological relevance is unknown and might differ between different sample preparations [50, 55]. To overcome this issue, the number of identified features, annotated in accordance with the metabolomics standard initiative (MSI), is commonly compared with the intention to better reflect the true number of molecules detected [56]. Another recently proposed strategy is to complement the total feature count by a subsequent SIRIUS analysis resulting in putatively characterized compound classes (MSI level 3 annotation) and applying the number of compounds classified as OAD (organic acids and derivatives) as an additional benchmark criterion for metabolomics analysis [46]. Figure 2A illustrates the general ClassyFire SuperClass distribution of organic compounds. Across all sample preparation procedures, OAD accounted for the largest proportion of compounds detected, followed by LLLS and organic oxygen compounds. In alignment with the total feature count evaluation, ME 400 also resulted in the highest number of compounds classified as OAD (141). Contrary to that, the number of OAD was higher for the MTBE-based procedure (96) compared to ME 700 (81), underlining the aforementioned limitations of sole feature count comparison (Table S8). Reproducibility was assessed by comparing the CV of average intensity normalized peak areas. For a sample preparation procedure-specific reproducibility assessment, the number and proportion of features with CV < 20% were considered for each independent feature list. As illustrated in Fig. 3A, the absolute number of features with a CV < 20% was higher for ME 400 (283) compared to ME 700 (223), but the proportions were similar with 27% and 25%, respectively. However, the MTBE-based procedure yielded a notably decreased number (121) and proportion of features with a CV < 20% of 13%. Additionally, for direct comparison, the CV distribution of selected features from a combined feature list across all sample preparation procedures (as described in Section “Metabolomics analysis”) is considered in Fig. 3B. The MTBE-based procedure revealed a significantly lower reproducibility of selected features (median CV = 31%) compared to ME 700 (median CV = 25%) and ME 400 (median CV = 21%).

Among the tested sample preparation procedures, ME 400 resulted in the highest total feature count and number of detected OAD as well as the best reproducibility. The higher number of detected OAD and total feature count using ME 400, compared to ME 700, was in accordance with the study by Muehlbauer et al., which reported that a reduction in bead size had a beneficial effect on the recovery of certain polar metabolites such as amino acids [23]. Furthermore, the results are in concordance with a previously published study from Brockbals et al., which evaluated different extraction solvent mixtures for postmortem tissue extraction using time-of-flight MS [46]. They also reported a similar feature count and number of OAD detected, but lower reproducibility for the MTBE-based procedure compared to the applied ME. Besides the properties of the extraction solvent mixture itself, increased variability of the MTBE-based procedure might be attributed to incomplete redistribution processes of compounds between the upper organic and lower aqueous phases. Even more important, an inconsistent separation of the two phases prior to evaporation could be a major contributor to the observed variability of the MTBE-based procedure. This particularly affects the metabolomics analysis, since the small volume of the lower aqueous phase requires careful pipetting during phase separation. Overall, the monophasic extraction in combination with 400-nm unmodified silica beads can be recommended for a comprehensive and consistent metabolomic profiling of HepG2 cells.

### Lipidomics

The suitability of the sample preparation procedures for lipidomics analysis was benchmarked complementary to Section “Metabolomics analysis.” Accordingly, the ability to comprehensively describe the lipidome was first assessed on the basis of the size of the total feature count. The MTBE-based procedure resulted in the highest total feature count in both positive (6483 ± 84) and negative (446 ± 3) ionization mode as shown in Fig. 1B. In both polarities, ME 700 showed a higher total feature count compared to ME 400 (Fig. 1B). Subsequently, the total feature count was complemented by the number of compounds classified as LLLS throughout SIRIUS analysis. For the MTBE-based procedure, the results were consistent as it also provided the highest number of LLLS (1591) (Table S8). However, in contrast to the total feature count evaluation, ME 400 provided a higher number of LLLS compared to ME 700 with 1453 and 1234, respectively (Table S8). Additionally, the ClassyFire class distribution of LLLS was assessed, acknowledging its conceptual differences from the LIPID MAPS classification system for lipids [57–59]. Figure 2B illustrates a similar ClassyFire class distribution of LLLS for all sample preparations, with fatty acyls being the most abundant group followed by glycerophospholipids. Reproducibility evaluation was performed accordingly to Section “Metabolomics analysis.” Figure 3C illustrates the number and proportion of features with CV < 20% assessed from independent feature lists. ME 400 provided a higher number and proportion of features with a CV < 20% than ME 700. However, the MTBE-based procedure achieved the best results, exhibiting 1373 features with a CV < 20%, which corresponds to a proportion of 21%. This was also consistent with the reproducibility of selected features from a combined feature list illustrated in Fig. 3D. Statistical analysis indicates a significantly lower median CV of selected features for the MTBE-based procedure (19%) in contrast to ME 400 (22%) and ME 700 (23%). Overall, the MTBE-based procedure showed improved performance compared to ME 400 and ME 700 for all assessed criteria regarding the lipidomics analysis. The suitability of the MTBE-based procedure for sole lipidomics analysis was thoroughly described in literature, since it was initially developed as an alternative for established extractions for comprehensive lipid profiling [21, 60–62]. Furthermore, these findings align with recent multiomics studies, which reported high reproducibility and broad lipid coverage for the MTBE-based solvent mixtures for the extraction of various matrices, such as biofluids, cells, and tissues [18, 20, 46, 63]. However, it is important to note that ME 400 and ME 700 did not exhibit any lipid class-specific bias compared to the MTBE-based procedure. As shown in Fig. 2B, the proportions of fatty acyls, glycolipids, glycerophospholipids, and sphingolipids are consistent across all sample preparation procedures. Consequently, ME 400 and ME 700 were appropriate in terms of qualitative lipidome representation using the analytical setting of this study. Compared with previous work, the implementation of nano-LC-IMS-HRMS/MS increased the analytical depth, as the number of compounds classified as LLLS exceeded the number of lipids identified in mouse adipocytes [23]. However, this comparison should be interpreted with caution, since different cell types and distinct computational tools for lipid annotation were used.

### Proteomics

**Fig. 4:**
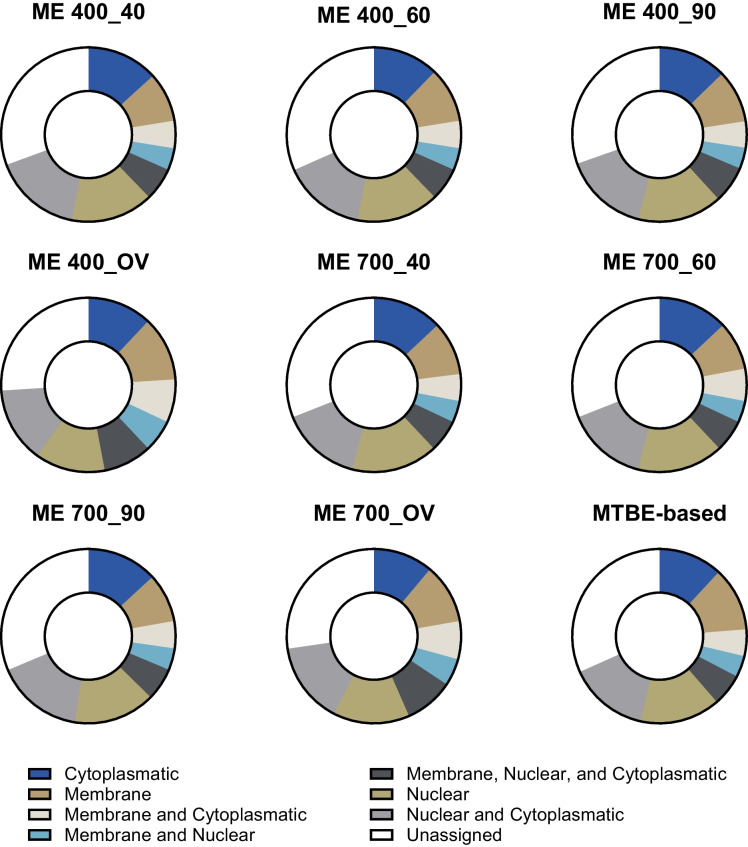
Comparison of Gene-Ontology (GO) cellular component category (membrane GO 0016020; nucleus GO 0005634; cytoplasm GO 0005737) distribution for proteins identified in all replicates of the associated sample preparation procedure. Donut charts represent the relative proportion of the possible GO-Term combinations for ME 400, ME 700, and MTBE-based, 40 (40 min), 60 (60 min), 90 (90 min), and OV (overnight, 840 min)

For the evaluation of the proteomics data, the sample preparation procedures were first investigated in terms of the number of identified peptides and proteins reflecting the comprehensiveness of proteomic profiling. Across all sample preparation procedures, a higher number of peptide identifications consistently corresponded to a higher number of protein identifications, as shown in Fig. S1A and Fig. 1C. The MTBE-based procedure, consisting of an overnight digestion of the interphase pellet, showed the highest number of identified proteins (5224 ± 232), followed by ME 700_40 (4765 ± 182). Moreover, in comparison to ME 700_40, no remarkable difference in the number of identified proteins was observed for ME 700_60 (4530 ± 182) and ME 700_90 (4480 ± 352), which are accelerated on-bead digestions with a diminished enzyme-to-protein ratio (Fig. 1C). In contrast, the overnight on-bead digestions with trypsin resulted in significantly decreased identifications, with 3603 ± 472 and 3210 ± 319 proteins being identified for ME 700_OV and ME 400_OV, respectively (Fig. 1C). The sequence coverage is an additional parameter for the evaluation of confidence and completeness of protein identification. For most proteins, a higher sequence coverage indicates a more reliable identification, as it suggests that multiple regions of the protein were successfully analyzed [64]. However, this does not always hold true for hydrophobic proteins, e.g., membrane proteins, due to inefficient denaturation and accessibility for tryptic digestion, among others [65]. The MTBE-based procedure and the accelerated on-bead digestions showed similar average sequence coverages of proteins identified across every sample preparation procedure and replicate (Fig. S1B). In alignment with the aforementioned results, the average sequence coverage was lowest for ME 700_OV and ME 400_OV with 13% and 12%, respectively (Fig. S1B). Missed cleavage (MC) rates are commonly considered using tryptic digestions, as they reflect the proteolytic cleavage efficiency under given experimental conditions. Figure S1C demonstrates that the overnight on-bead digestions ME 700_OV (MC_0_ ≈ 88%, MC_1_ ≈ 11%, MC_2_ ≈ 1%) and ME 400_OV (MC_0_ ≈ 88%, MC_1_ ≈ 12%, MC_2_ ≈ 1%) provided the highest proportion of fully digested proteins, followed by the MTBE-based procedure (MC_0_ ≈ 78%, MC_1_ ≈ 20%, MC_2_ ≈ 2%), whereas the missed cleavage rate was generally higher for the accelerated on-bead digestions using rapid trypsin. Lower rates generally suggest a more complete digestion and are associated with improved peptide identification and proteome coverage, as previously reported [66, 67]. While the missed cleavage rate is primarily of computational relevance, and in the present study, a higher rate of missed cleavages was not coherent with a reduced number of protein identifications for the on-bead digestions, this parameter was ranked lower in the overall evaluation for these proteomic workflows. To assess potential differences in subcellular protein representation, particularly between on-bead digestions and digestions from the interphase pellet, the distribution of the reproducibly identified proteins across the considered Gene-Ontology (GO) cellular component categories (membrane GO 0016020; nucleus GO 0005634; cytoplasm GO 0005737) was analyzed. Figure 4 illustrates that all sample preparation procedures recover membrane, nuclear, and cytoplasmic proteins in a comparable manner. Considering the applied variations of the ME procedures, the results of the proteomic analysis indicate that the reduction of bead diameter from 700 to 400 nm was not beneficial, since all evaluated parameters showed highly similar results for the corresponding digestion conditions only differing in bead type. This is in accordance with previous studies, which have reported that specific bead properties had only a minor impact on the unspecific binding of proteins on the bead surface [8, 23]. However, a reduction in the enzyme-to-protein ratio from 1:10 to 1:25 is more sustainable, since it is feasible without a concomitant loss of proteomic depth, and the additional extension of the digestion time from 60 to 90 min is not required. Although the MTBE-based procedure has proven to be advantageous regarding the proteomics depth, it was associated with more complex sample handling due to the resuspension step and the extended digestion time. Consequently, ME-accelerated on-bead digestion was selected as the preferred method for proteomic sample preparation of plated HepG2 cells.

### Summary and limitations

Comprehensive multiomics profiling requires careful selection of analytical techniques and sample preparation strategies to appropriately address the characteristics of each omics layer. Due to the high molecular diversity encountered across the different layers, complementary analytical platforms were utilized in this study. Specifically, orbitrap-based LC-HRMS/MS was employed for metabolomics analyses, taking advantage of its high mass accuracy and resolution for small-molecule applications. In contrast, nano-LC-IMS-HRMS/MS was applied for proteomics and lipidomics, where the advantages in speed and selectivity arising from the use of the parallel accumulation–serial fragmentation scan mode, combined with the benefits provided by the ion mobility dimension during downstream data processing, outweigh other considerations in terms of molecular coverage. Following the adaptation to this specific setup, the current study aimed to compare the suitability of a monophasic bead-based approach with a biphasic MTBE-based sample preparation procedure for integrated multiomics analysis of plated HepG2 cells. The evaluation of the sample preparation procedures demonstrated that their suitability is highly dependent on the investigated molecular class. For metabolomic profiling, ME 400 resulted in the most comprehensive and reproducible feature detection, whereas the MTBE-based procedure showed lowest metabolome coverage and highest variability. In contrast, the MTBE-based procedure was most suitable for lipidomic analysis, which is in line with literature where it has been shown to be valid for robust lipidomic assessment with broad lipid coverage [46, 63]. The proteomic results were mostly unaffected by the accelerated on-bead digestion conditions applied, whereas overnight on-bead digestion resulted in decreased proteomic depth. While the MTBE-based procedure provided the highest number of identified peptides and proteins, it was disadvantageous and less sustainable regarding the number of sample preparation steps, handling complexity, and digestion time. In general, an ideal sample preparation procedure for untargeted analysis should be unselective and reproducible while still being straightforward and rapid with a minimal number of manual steps [68]. However, due to the wide variety of molecules under investigation, strongly differing in physiochemical properties, a perfect sample preparation procedure is hard to achieve. Thus, a combination of several sample preparation methods would be required. This however is unfeasible in most cases due to the considerable effort involved. Therefore, a compromise considering both performance and handling complexity should be made, and ME 400_60 might be recommended. ME generally showed improved ease of handling as no time-consuming and error-prone phase separation or protein resolubilization steps were required.

Overall, these findings align with those of the previous study by Muehlbauer et al., who first introduced the bead-enabled accelerated monophasic multiomics method as a fast and simple alternative for the preparation of various sample types. While its performance has been experimentally benchmarked against other protocols particularly for human plasma, comparisons for other sample types have predominantly relied on literature-based evaluations rather than direct experimental testing. The present study expands this knowledge by demonstrating that the ME procedures are also suitable for integrative multiomics analysis of plated HepG2 cells. Furthermore, the original protocol was refined through optimized sample preparation and partial transfer of the analytical setup, generating methodological improvements. Particularly, the lipidomics analysis benefited from the implementation of nano-LC-IMS-HRMS/MS, which improved sustainability and increased analytical depth. The use of 400 nm instead of 700-nm beads has proven advantageous, especially for the metabolomics analysis. Finally, reducing the enzyme-to-protein ratio of rapid trypsin, while slightly extending the incubation time, made the digestion more cost-effective and contributed to a more streamlined workflow.

Nonetheless, the result of the present multiomics study is limited. While many proteomics and lipidomics studies only use one chromatographic system, sophisticated metabolomics analysis often requires to apply both reversed-phase chromatography and HILIC to enhance the representation of chemically diverse metabolite classes [68]. However, in this multiomics study, only HILIC was applied due to feasibility considerations, particularly since it was expected that certain apolar small molecules would also be captured in the lipidomics analysis performed using reversed-phase chromatography. Future steps in workflow evolution might be the reconstitution solvent for the used matrix and individual analytical settings [50]. Moreover, while nano-LC was successfully implemented for proteomics and lipidomics analyses, its application to metabolomics was not considered in this study. This decision reflects current technical limitations, particularly the lack of robust and commercially available HILIC columns compatible with nanoflow conditions. The reproducibility and long-term stability of existing nano-HILIC solutions remain insufficient for untargeted metabolomics applications. Consequently, HILIC under conventional flow regimes was retained for the analysis of polar metabolites. As the availability and performance of nano-HILIC technologies improve, future implementations may extend nano-LC applicability to untargeted metabolomics workflows as well. This analytical setup could potentially enhance the detectability of low-abundant or structurally similar metabolites.

## Conclusion

The present study transferred, adapted, and evaluated an untargeted workflow for the simultaneous metabolomics, lipidomics, and proteomics analysis of HepG2 cells using LC-HRMS/MS and LC-IMS-HRMS/MS. Among the considered sample preparation protocols, a monophasic extraction procedure with an adapted bead diameter of 400 nm, a short digestion time of 60 min, and a reduced enzyme-to-protein ratio (1:25) of rapid trypsin was found to be the most suitable approach. While other protocols may have performed better in single aspects, the chosen sample preparation allowed for a reproducible, streamlined, and cost-efficient implementation of a multiomics workflow, with respect to the specific experimental conditions and instrumental setup.

## Supplementary Information

Below is the link to the electronic supplementary material.

Supplementary Material 1 (PDF 254 KB)

## Data Availability

All data supporting the findings of this study are available upon request from the corresponding author. Proteomics data have been deposited to PRIDE repository (ProteomeXchange Consortium), the dataset identifier is available upon request from the corresponding author.
