# Supplementary material for: Optimizing multiomics sample preparation: comparative evaluation of extraction protocols for HepG2 cells
Source: Anal Bioanal Chem. 2025 Nov 19;418(3):813–27. doi: 10.1007/s00216-025-06235-x (PMC12890986; doi:10.1007/s00216-025-06235-x)
Supplement: Supplementary file 1 — Supplementary Material 1 (PDF 254 KB) [file 216_2025_6235_MOESM1_ESM.pdf]

Optimizing Multiomics Sample Preparation: Comparative Evaluation of Extraction Protocols for HepG2 Cells

Tilman F. Arnst, Selina Hemmer, Claudia Fecher-Trost, Lea Wagmann, and Markus R. Meyer

Experimental and Clinical Toxicology and Pharmacology, Center for Molecular Signaling (PZMS), PharmaScienceHub (PSH), Saarland University, Homburg, Germany

*Corresponding author

🖂 Markus R. Meyer

markus.meyer@uni-saarland.de

1.1. Bead preparation

For the preparation of the bead stock solution, the amount of unmodified silica beads SeraSil-Mag 700 or SeraSil-Mag 400 required for a bead-to-protein ratio of 10:1 was estimated based on the BCA assay results. Consequently, 1760 µL bead solution were washed twice with nanofiltered water using a magnetic rack. Afterwards, the beads were resuspended in 1400 µL of nanofiltered water. Bead stock solutions were prepared on the day of sample preparation and stored at room temperature until usage.

1.2. BCA assay

Total protein amount was estimated with a Pierce™ BCA protein assay kit (Thermo Fisher Scientific, Dreieich, Germany), according to the manufacturer's instructions, using a 1:50 dilution of three wells per plate.

1.3. Sirius processing parameters for metabolomics

The following computational settings have been used for metabolomic datasets: Instrument: Orbitrap, Filter by isotope pattern: on; MS2 accuracy: 5 ppm; MS/MS isotope scorer: ignore; Candidates stored: 10; Min candidates per ionization stored: 1; Fix formula for detected lipid: on; Fallback Adducts: all; Molecular formula generation: De novo + bottom up; Perform de novo below *m/z*: 400; Apply element filter to: De novo; Allowed elements: H, C, N, O, P; Autodetect: B, S, Cl, Se, Br; ZODIAC: activated; CSI:FingerID and CANOPUS: activated; Score threshold: on; PubChem as fallback: on; Confidence mode: APPROXIMATE; Search DBs: Biocyc, Blood Exposome, CHEBI, COCONUT, DSSTOX, FooDB, GNPS, HMDB, HSDB, KEGG, KNApSAcK, LOTUS, LipidMaps, Maconda, MeSH, MiMeDB, NORMAN, PubMed, PubChem safety and toxic.

1.4. Sirius processing parameters for lipidomics

For the analysis in SIRIUS, features with *m/z* > 850 were also processed. Molecular formulae generated in Metaboscape were not considered. The following computational settings have been used for lipidomic datasets: Instrument: Q-TOF, Filter by isotope pattern: on; MS2 accuracy: 5 ppm; MS/MS isotope scorer: ignore; Candidates stored: 10; Min candidates per ionization stored: 1; Fix formula for detected lipid: on; Fallback Adducts: all; Molecular formula generation: De novo + bottom up; Perform de novo below *m/z*: 400; Apply element filter to: De novo; Allowed elements: H, C, N, O, P; Autodetect: B, S, Cl, Se, Br; ZODIAC: activated; CSI:FingerID and CANOPUS: activated; Score threshold: on; PubChem as fallback: on; Confidence mode:

APPROXIMATE; Search DBs: Blood Exposome, GNPS, HMDB, HSDB, KEGG, LipidMaps, PubChem drug; PubChem safety and toxic.

1.7 Generation of a HepG2 proteome in-house peptide spectral library – sample preparation, analysis and processing

HepG2 cells grown in a cell culture (75 cm^2) were resuspended with 500 µl RIPA lysis buffer (150 mM NaCl, 50 mM Tris HCl pH 8, 5 mM EDTA, 1% Nonidet P40 (v/v), 0.1% SDS (w/v), 0.5% Na-deoxycholate (*w:v*) which was precooled at 6 °C, with a freshly added protease inhibitor cocktail (Roche, Mannheim, Germany). The cells were lysed using ultrasound sonification (30 sec pulsed intervals, 50% amplitude, Bandelin, Berlin, Germany), sheared 10 times (24G gauge needle) and incubated for 30 minutes at 4°C on a shaker. The suspension was ultracentrifuged at 100.000 *x* g at 6 °C for 1h (Beckmann Coulter Optima^{TM} MAX-E ultracentrifuge). The protein amount was determined and 100 µg total protein was mixed with 2x denaturing buffer (SDS 8 % (*w:v*) TRIS pH 6.8 120 mM, bromophenol blue 0.01% (*w:v*), glycine 20% (*v:v*), β-mercaptoethanol 10% (*v:v*)) and denatured for 20 min a 60 °C. Proteins were separated on a 4-12% gradient gel (Bolt, ThermoFisher, Karlsruhe, Germany) at 200 V using MOPS-SDS electrophoresis buffer. Proteins were fixed by incubation with 40% ethanol/ 10% acetic acid for 30 min, the gel was washed three times with demineralized water and proteins visualized with a colloidal Coomassie staining (0.12% (*w:v*) Coomassie G250, 10% (*w:v*) ammonium sulphate, 10% (*v:v*) phosphoric acid, 20% (*v:v*) methanol in water) for 1 h. After three times washing with demineralized water for 5 min eight bands per sample lane were cut and alternately washed two times with solution A (50 mM NH_4HCO_3) and B (50 mM NH_4HCO_3 and 50% (*v:v*) acetonitrile) for 10 min on a shaker. Disulfide bridges were reduced with 10 mM dithiothreitol (in wash solution A) for 30 min at 56 °C, before carbamidomethylation with 5 mM iodoacteamide in wash solution A was done for 30 min in the dark. Gel bands were washed alternately with wash solutions A and B again. After drying the gel bands in a vacuum centrifuge, they were incubated overnight with 15 µl porcine trypsin (10 ng/µl, Promega) at 37 °C. Tryptic peptides were extracted twice after incubation with 50 µl extraction buffer (2.5% formic acid, 50% acetonitrile) in an ultrasonic bath. The combined supernatants were dried in a vacuum centrifuge and resuspended in 20 µl 0.1% (*v:v*) formic acid. Proteomics analysis was performed on a nanoElute (Bruker Daltonics) coupled with a CaptiveSpray source to a timsTOF Pro 2 (Bruker Daltonics) [39]. Pre-acquisition mass and ion mobility calibration was performed as before. Six µL sample were loaded onto a Acclaim^{TM} PepMap^{TM} 100 (100 µm x 2 cm, TF Scientific) and separated using a PepSep® Column (25 cm x 150 µm, Bruker Daltonics). Peptide elution was performed using eluent A (water with 0.1% formic acid, *v:v*) and

eluent B (ACN with 0.1% formic acid, *v:v*). A detailed description regarding the gradient program and the mass spectrometry settings using data dependent acquisition (DDA) mode are given in Table S6 and Table S7. Subsequently, data processing was performed using PEAKS Studio (version 10.6 build 20201221). Database search was carried out with PEAKS DB via monoisotopic precursor mass search with a parent mass error tolerance of 25.0 ppm and fragment mass error tolerance of 0.05 Da. Identification was conducted using UniProt Homo sapiens database (20354 sequences, Version November 2023) as reference database. For peptide identification, three missed cleavages were allowed, a carbamidomethylation of cysteines was used as a static modification, oxidation of methionine residues, acetylation of protein N-termini and deamidation of asparagine and glutamine residues were allowed as variable modifications. For protein identification at least two unique peptides with a false discovery rate (FDR) < 1% were required. Subsequently, peptide identifications were used to generate a spectral library within PEAKS Studio.

Table S1. Overview of number of replicates used for each extraction and digestion condition for metabolomics[1], lipidomics[2], and proteomics analysis[3]. ME (monophasic extraction), 400 (400 nm beads), 700 (700 nm beads), 40 (40 minutes), 60 (60 minutes), 90 (90 minutes), OV (overnight, 840 minutes), MTBE-based (methyl-*tert*-butyl ether-based).

Extraction and Digestion conditions	Replicates (n)
ME 400 [1,2]	16
ME 700 [1,2]	16
ME 400_40 [3]	4
ME 400_60 [3]	4
ME 400_90 [3]	4
ME 400_OV [3]	4
ME 700_40 [3]	4
ME 700_60 [3]	4
ME 700_90 [3]	4
ME 700_OV [3]	4
MTBE-based [1,2,3]	8

Table S2. Final concentrations of stable isotope-labeled internal standards in extraction solvents.

Internal standard	Concentration, µmol/L
Cytosine-d_2	9
L-carnitine-d_9	59
L-arginine-d_7	55
Kynurenic acid-d_5	5
L-tryptophan-d_5	48
Thymidine-d_4	2
Stearic acid-^{13}C	175
1-Palmitoyl-d_9-2-palmitoyl-sn-glycerol	2

Table S3. Thermo Fisher Q Exactive Plus conditions for metabolomics analysis.

**Parameter**	**Settings**
Ionization mode	positive or negative
Sheath gas	60 AU
Auxiliary gas	10 AU
Sweep gas	3 AU
Spray voltage	3.5 kV
Heater temperature	320 °C
Ion transfer capillary temperature	320 °C
S-lens RF level	60
Full scan data acquisition	
Positive resolution	35,000
Scan range	50 to 750 *m/z*
Automatic gain control	1e6
Maximum injection time	120 ms
Microscans	1
Data dependent MS^2	
Resolution	17,500
Isolation window	1.0 *m/z*
AGC target	2e5
Maximum injection time	250 ms
High collision dissociation cell collision energy	10, 20, 40
Loop count	5
Minimum AGC target	1e3
Exclude isotopes	On
Spectrum data type	Profile

Table S4. Bruker timsTOF Pro 2 conditions for lipidomics analysis in positive and negative ionization mode.

**Parameter**	**Settings**	
Polarity	positive	negative
Scan Mode	PASEF	PASEF
Mass range	100 – 1350 *m/z*	100 – 1350 *m/z*
$1/k_0$ range	0.55 – 1.90 Vs/cm^2	0.55 – 1.74 Vs/cm^2
ICC	On	On
Target	7.5 Mio	7.5 Mio
Ramp time	100.0 ms	100.0 ms
Lock duty cycle to 100 %	on	on
Source		
Source	CaptiveSpray	CaptiveSpray
Capillary	1500 V	1500 V
NanoBooster	0.20 Bar	0.20 Bar
Dry Temp	180 °C	180 °C
Dry Gas	3.0 L/min	3.0 L/min
Tune General		
Deflection 1 delta	80.0 V	-80.0 V
Funnel 1 RF	250.0 Vpp	250.0 Vpp
Funnel 2 RF	250.0 Vpp	250.0 Vpp
isCID Energy	0.0 eV	0.0 eV
Multipole RF	200.0 Vpp	200.0 Vpp
Ion Energy	5.0 eV	5.0 eV
Low Mass	150 *m/z*	150 *m/z*
Collision Energy	10.0 eV	10.0 eV
Collision RF	1100.0 Vpp	1100.0 Vpp
Transfer Time	65.0 µs	65.0 µs
Pre Pulse Storage	5.0 µs	5.0 µs
High Sensitivity Detection	Off	Off
Stepping	Off	Off
Tune TIMS		
Dt1	-20.0 V	20.0 V
Dt2	-120.0 V	120.0 V
Dt3	80.0 V	-80.0 V
Dt4	100.0 V	-100.0 V
Dt5	0.0 V	0.0 V
Dt6	100.0 V	-100.0 V
Funnel 1 RF	500.0 Vpp	500.0 Vpp
Collision Cell in	220.0 V	-220.0 V
PASEF		
TIMS Stepping	Off	Off
No. of PASEF MS/MS scans	2	2
Target Intensity	4000	4000
Intensity Threshold	100	100
Charge Range	0 – 1	0 – 1
Active Exclusion	On	On
Release after	0.10 min	0.10 min
Reconsider Precursor	On	On
Current/Previous Intensity	2.00	2.00
Isolation Width Range	2.00 *m/z*	2.00 *m/z*
Energy Range	30.0 to 30.0 eV	-35.0 to -35.0 eV

Table S5. Bruker timsTOF Pro 2 conditions for proteomics analysis.

**Parameter**	**Settings**
Polarity	Positive
Scan Mode	dia-PASEF
Mass range	100 – 1700 *m/z*
$1/k_0$ range	0.85 – 1.30 Vs/cm^2
Rolling Average	On
Rolling Average No.	10
ICC	Off
Target	2.0 Mio
Ramp time	100.0 ms
Lock duty cycle to 100 %	on
Accumulation Time	2.0 ms
Source	CaptiveSpray
Capillary	1500 V
NanoBooster	0.20 Bar
Dry Temp	180 °C
Dry Gas	3.0 L/min
Tune General	
Deflection 1 delta	70.0 V
Funnel 1 RF	300.0 Vpp
Funnel 2 RF	200.0 Vpp
isCID Energy	0.0 eV
Multipole RF	500.0 Vpp
Ion Energy	5.0 eV
Low Mass	200.00 *m/z*
Collision Energy	10.0 eV
Collision RF	1500.0 Vpp
Transfer Time	60.0 µs
Pre Pulse Storage	12.0 µs
High Sensitivity Detection	Off
Stepping	Off
Tune TIMS	
Dt1	-20.0 V
Dt2	-160.0 V
Dt3	110.0 V
Dt4	110.0 V
Dt5	0.0 V
Dt6	55.0 V
Funnel 1 RF	475.0 Vpp
Collision Cell in	300.0 V

Table S6. Reversed-phase chromatography conditions for proteomics analysis for peptide library generation, including elution mode, column temperature, sample loading volume, sample loading pressure, flow rate and chromatographic gradient. Seperation was performed using a AcclaimTM PepMapTM 100 (100 µm x 2 cm, TF Scientific) and a PepSep® Column (25 cm x 150 µm, Bruker Daltonics). Peptide elution was performed using eluent A (water with 0.1% formic acid, *v:v*) and eluent B (ACN with 0.1% formic acid, *v:v*).

**Parameter**	**Settings**
Elution mode	Two column separation
Column temperature	50.0 °C
Sample loading volume	4 pick-up volumes + 2 µL
Sample loading pressure	210.5 bar
Flow rate	0.80 µL/min
**Time, min**	**Composition, %B**
0.00 - 120.00	2.0 - 35.0
120.00 - 120.50	35.0 - 95.0
120.50 - 133.88	95.0

Table S7. Bruker timsTOF Pro 2 conditions for proteomics analysis for peptide library generation.

**Parameter**	**Settings**
Polarity	Positive
Scan Mode	PASEF
Mass range	100 – 1700 *m/z*
$1/k_0$ range	0.60 – 1.60 Vs/cm^2
Rolling Average	On
Rolling Average No.	10
ICC	Off
Target	2.0 Mio
Ramp time	100.0 ms
Lock duty cycle to 100 %	on
Accumulation Time	2.0 ms
Source	CaptiveSpray
Capillary	1600 V
NanoBooster	Off
Dry Temp	180 °C
Dry Gas	3.0 L/min
Tune General	
Deflection 1 delta	70.0 V
Funnel 1 RF	300.0 Vpp
Funnel 2 RF	200.0 Vpp
isCID Energy	0.0 eV
Multipole RF	500.0 Vpp
Ion Energy	5.0 eV
Low Mass	200.00 *m/z*
Collision Energy	10.0 eV
Collision RF	1500.0 Vpp
Transfer Time	60.0 µs
Pre Pulse Storage	12.0 µs
High Sensitivity Detection	Off
Stepping	Off
Tune TIMS	
Dt1	-20.0 V
Dt2	-160.0 V
Dt3	110.0 V
Dt4	110.0 V
Dt5	0.0 V
Dt6	55.0 V
Funnel 1 RF	450.0 Vpp
Collision Cell in	300.0 V

Table S8 Number of compounds classified as organic acids and derivatives (OAD) as benchmark criterium for metabolomics analysis using hydrophilic interaction chromatography. Number of compounds classified as lipids and lipids-like species (LLLS) as benchmark criterium for lipidomics analysis using reversed phase chromatography. Prerequisites for consideration were a ZODIAC score >800 and a ClassyFire superclass probability >700 using the CANOPUS and CSI:FingerID algorithm within SIRIUS (Version 6.1.0). ME (monophasic extraction) 400 (400 nm beads), ME 700 (700 nm beads), and MTBE (methyl-tert-butyl ether)-based sample preparation.

Sample preparation	OAD	LLLS
ME 400	141	1453
ME 700	81	1234
MTBE-based	96	1591

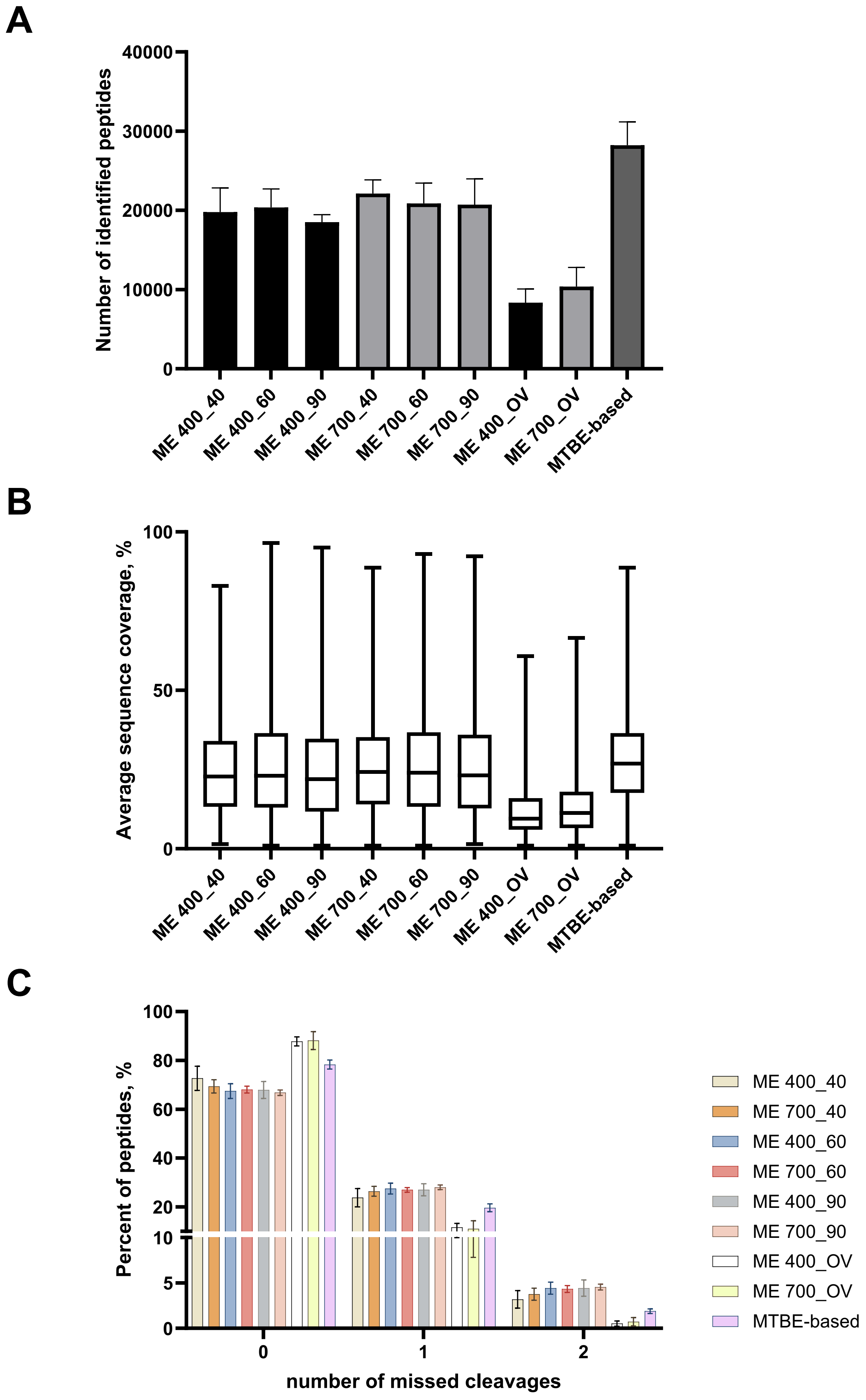
A
Number of identified peptides
40000
30000
20000
10000
0
ME 400_40
ME 400_60
ME 400_90
ME 700_40
ME 700_60
ME 700_90
ME 400_OV
ME 700_OV
MTBE-based
B
Average sequence coverage, %
100
50
0
ME 400_40
ME 400_60
ME 400_90
ME 700_40
ME 700_60
ME 700_90
ME 400_OV
ME 700_OV
MTBE-based
C
Percent of peptides, %
100
80
60
40
20
10
5
0
0
1
2
number of missed cleavages
ME 400_40
ME 700_40
ME 400_60
ME 700_60
ME 400_90
ME 700_90
ME 400_OV
ME 700_OV
MTBE-based

Fig. S1: (A) Histograms with number of identified peptides for ME (monophasic extraction) 400 (400 nm beads), ME 700, and MTBE (methyl-tert-butyl ether)-based, 40 (40 minutes), 60 (60 minutes), 90 (90 minutes), and OV (overnight, 840 minutes), MTBE-based (n=8), all others (n=4) (B): Box-plots, with the average sequence coverage in percent for proteins identified in all samples. Boxes represent the interquartile range (IQR), from the first quartile (25th percentile) to the third quartile (75th percentile). Medians (50th percentiles) are shown as horizontal lines within the boxes. Whiskers indicate the minimum and maximum values. (C) Histograms with the number of missed cleavages (0, 1, or 2) in percent based on the total number of identified peptides for each sample preparation procedure. Bars and error bars indicate mean ± SD (standard deviation).
